# Mint3 depletion-mediated glycolytic and oxidative alterations promote pyroptosis and prevent the spread of *Listeria monocytogenes* infection in macrophages

**DOI:** 10.1038/s41419-021-03691-y

**Published:** 2021-04-14

**Authors:** Takayuki Uematsu, Kohsuke Tsuchiya, Noritada Kobayashi, Motoharu Seiki, Jun-ichiro Inoue, Shuichi Kaneko, Takeharu Sakamoto

**Affiliations:** 1 Biomedical Laboratory, Division of Biomedical Research, Kitasato University Medical Center, Arai, Kitamoto, Saitama Japan; 2 Division of Immunology and Molecular Biology, Cancer Research Institute, Kanazawa University, Kakuma-machi, Kanazawa, Ishikawa Japan; 3 Division of Cancer Cell Research, Institute of Medical Science, The University of Tokyo, Shirokanedai, Minato-ku, Tokyo, Japan; 4 Division of Cellular and Molecular Biology, Institute of Medical Science, The University of Tokyo, Shirokanedai, Minato-ku, Tokyo, Japan; 5 Department of System Biology, Institute of Medical, Pharmaceutical and Health Sciences, Kanazawa University, Takara-machi, Takara-machi, Kanazawa, Ishikawa Japan

**Keywords:** Cell death, Infection

## Abstract

*Listeria monocytogenes* (LM) infection induces pyroptosis, a form of regulated necrosis, in host macrophages via inflammasome activation. Here, we examined the role of Mint3 in macrophages, which promotes glycolysis via hypoxia-inducible factor-1 activation, during the initiation of pyroptosis following LM infection. Our results showed that Mint3-deficient mice were more resistant to lethal listeriosis than wild-type (WT) mice. Additionally, the mutant mice showed higher levels of IL-1β/IL-18 in the peritoneal fluid during LM infection than WT mice. Moreover, ablation of Mint3 markedly increased the activation of caspase-1, maturation of gasdermin D, and pyroptosis in macrophages infected with LM in vitro, suggesting that Mint3 depletion promotes pyroptosis. Further analyses revealed that Mint3 depletion upregulates inflammasome assembly preceding pyroptosis via glycolysis reduction and reactive oxygen species production. Pharmacological inhibition of glycolysis conferred resistance to listeriosis in a Mint3-dependent manner. Moreover, Mint3-deficient mice treated with the caspase-1 inhibitor VX-765 were as susceptible to LM infection as WT mice. Taken together, these results suggest that Mint3 depletion promotes pyroptosis in host macrophages, thereby preventing the spread of LM infection. Mint3 may serve as a target for treating severe listeriosis by inducing pyroptosis in LM-infected macrophages.

## Introduction

*Listeria monocytogenes* (LM) is a Gram-positive short rod that is highly abundant in the intestinal tracts of ruminants. LM causes listeriosis, which is zoonotically transmitted through contaminated food such as dairy products. In pregnant women, bacteremia arising from the intestinal tract and the liver can infect the fetus and cause meningitis. Listeriosis is frequently observed in modern countries, such as the United States, Australia, and European countries, which consume large quantities of dairy products^1,2^.

LM serves as an excellent model of an intracellular parasite for immunology-related studies^3,4^. Upon internalization into host cells, LM escapes from the primary phagosomes and proliferates in the cytoplasm, where it evades host immunity by expressing various pathogenic factors represented by listeriolysin O (LLO). The host mounts an innate immune response to LM by inducing Th1-type cellular immunity. Further, interferon-γ (IFN-γ) produced by Th1 cells activates macrophages, and LM remaining in the cytoplasm is killed by the action of reactive oxygen species (ROS) and nitric oxide (NO)^5^.

Bacteria-infected macrophages activate a suicidal-death program, called pyroptosis, which clears the field from bacterial growth and contributes to host defense^6^. LM infection also triggers pyroptosis^7,8^. Genomic DNA and LLO of LM activate AIM2- or NLRP3 inflammasomes^9,10^. Subsequently, caspase-1 is activated in an inflammasome-dependent manner^11,12^, and gasdermin D (GSDMD) is cleaved. The N-terminal fragment of GSDMD forms a polymeric pore on the cell membrane and induces pyroptosis^13^. Thus, pyroptosis contributes to preventing the expansion of LM infection, although the precise molecular mechanisms underlying the regulation of LM-induced pyroptosis in macrophages remain unknown.

The X11 family member Mint3 (also known as APBA3) activates hypoxia-inducible factor-1 (HIF-1) in the presence of oxygen by suppressing its inhibitor, FIH-1 (refs. ^14–18^). Mint3 requires the transmembrane protease MT1-MMP to bind and inhibit FIH-1 (refs. ^19,20^). Thus, Mint3-mediated HIF-1 activation is limited to cells such as macrophages and cancer cells that express MT1-MMP^21^. Mint3-deficient mice are viable and do not exhibit detectable macroscopic defects^22^. However, macrophages isolated from these mutant mice are defective in glycolytic ATP synthesis because of decreased HIF-1 activity. Mint3-deficient macrophages are also defective in cytokine production in response to lipopolysaccharide (LPS)^22,23^.

FIH-1 hydroxylates proteins other than HIF-1α, such as IκBα, which inhibits the nuclear factor-κB (NF-κB) signaling^24^. Thus, in macrophages, Mint3 inhibition of FIH-1 activity may alter the production of inflammatory cytokines and chemokines through the effect of FIH-1 on HIF-1 and NF-κB signaling. Furthermore, Mint3 contributes to the innate immune response to viral infection^25,26^ and the survival or growth of cancer cells^27–31^. Therefore, this study aimed to assess for the involvement of Mint3 in the pathogenesis of listeriosis and protection of the host against LM infection.

## Results

### Mint3 depletion attenuates severe listeriosis in mice

**Fig. 1:**
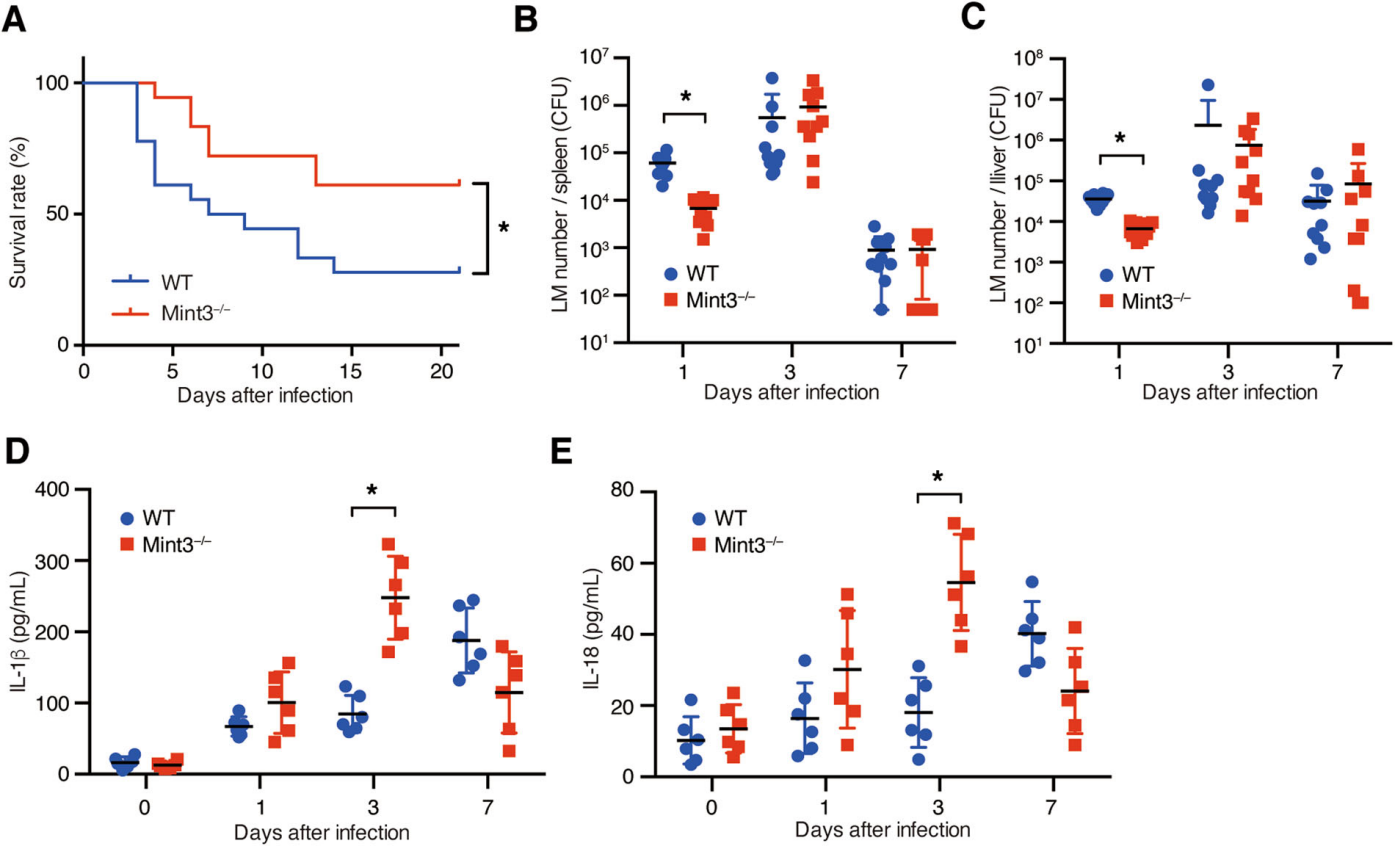
Loss of Mint3 attenuates severe listeriosis. **A** Survival rates of mice following infection with *Listeria monocytogenes* (LM). Both WT and *Mint3*^–/–^ mice (*n* = 18 per group) were infected via intraperitoneal injection of 2 × 10^5^ colony-forming units (CFUs) of LM, followed by the assessment of survival rates. Data are representative of two independent experiments. *P < 0.05 by the log-rank test. **B** Bacterial burdens in the spleen and **C** liver, counted on days 1, 3, and 7 after LM infection. **D** IL-1β and **E** IL-18 production in the peritoneal cavities of WT and *Mint3*^–/–^ mice. Data are presented as the means ± SEMs and are representative of two independent experiments. *P < 0.05 by the Mann–Whitney *U* test.

To determine whether Mint3 contributes to immune responses during LM infection, we first intraperitoneally injected a lethal dose of LM into wild type (WT: C57BL/6) and *Mint3*^–/–^ mice. LM-infected WT mice appeared visibly ill, as indicated by ruffled fur, slow movements, and >10% reduction of body weight, 4–10 days after infection (Supplementary Fig. 1), whereas *Mint3*^–/–^ mice appeared more active than WT mice. Consistent with their apparent activity, the final survival rate on day 21 after LM infection was significantly improved in *Mint3*^–/–^ mice (∼60%) compared with that in WT mice (∼30%; Fig. 1A). One day after LM infection, the bacterial burdens in the spleen (Fig. 1B) and liver (Fig. 1C) were significantly lower in *Mint3*^–/–^ mice than in WT mice. On the other hand, at later time points (3 and 7 days after LM infection), there was no significant difference in bacterial counts between the strains (Fig. 1B, C).

LM infection activates the AIM2/NLRP3 inflammasome, leading to the release of the cytokines interleukin-1β (IL-1β) and IL-18, which contribute to innate immunity^3,9–12,32^. We, therefore, determined the levels of IL-1β and IL-18 in peritoneal lavage fluid harvested from LM-infected mice. IL-1β (Fig. 1D) and IL-18 (Fig. 1E) levels were significantly increased in the peritoneal fluid of *Mint3*^–/–^ mice 3 days after LM infection. These findings indicate that Mint3 depletion attenuated severe listeriosis and reduced mortality. Furthermore, Mint3 deficiency led to increased production of IL-1β and IL-18, which may have contributed to antibacterial immunity.

### Mint3 depletion attenuates LM growth within macrophages

**Fig. 2:**
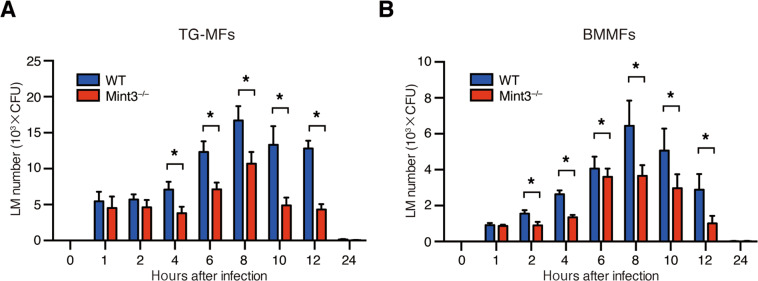
Mint3 deficiency attenuates LM growth within macrophages. **A** Thioglycolate-induced peritoneal macrophages (TG-MFs) and **B** bone marrow-derived macrophages (BMMFs) from WT or *Mint3*^–/–^ mice were infected in vitro with 10^5^ CFUs of LM (MOI = 1). The bacterial burden was determined 1, 2, 4, 6, 8, 10, 12, and 24 h after infection. Data are presented as the means ± SDs of triplicates. Data are representative of two independent experiments. *P < 0.05 by the Student’s *t*-test.

LM can initially survive and grow within macrophages, but later LM infection activates macrophages through the action of IFN-γ derived from Th1 cells, leading to the termination of infection^4^. Thus, macrophages play an important role in LM infection. Mint3 depletion affects macrophage function owing to reduced HIF-1 activity and ATP production via glycolysis^22,23^. Thus, we next examined whether Mint3 is required for LM infection and growth within macrophages. Thioglycolate-induced peritoneal macrophages (TG-MFs) and bone marrow-derived macrophages (BMMFs) from WT or *Mint3*^–/–^ mice were infected with LM in vitro, and then the intracellular bacterial burdens were measured. The intracellular invasion of LM was not affected, but the intracellular growth of LM was significantly suppressed in *Mint3*^–/–^ TG-MFs (Fig. 2A) and BMMFs (Fig. 2B). Thus, decreased LM growth within macrophages likely contributed to the attenuation of severe listeriosis in *Mint3*^–/–^ mice.

### Mint3 depletion leads to decreased ROS production and increased cell death in LM-infected macrophages

**Fig. 3:**
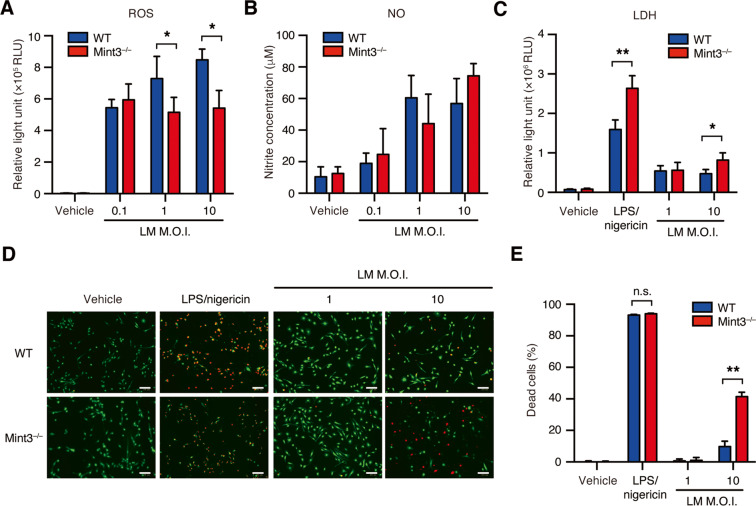
Mint3 deficiency leads to decreased reactive oxygen species (ROS) production and increased cell death in LM-infected macrophages. Production of **A** (ROS) and **B** nitric oxide (NO) in WT or *Mint3*^–/–^ BMMFs. BMMFs from WT or *Mint3*^–/–^ mice were infected in vitro with LM (MOI = 0.1, 1, or 10). The production of ROS and NO was measured by luminometry 8 h after LM infection and Griess assay 12 h after LM infection, respectively. Data are presented as the means ± SDs of triplicates and are representative of two independent experiments. *P < 0.05 by the Student’s *t*-test. **C–E** LM-infection-associated cell death of WT or *Mint3*^–/–^ BMMFs. As a positive control, BMMFs were primed for 6 h with LPS (50 ng/mL), followed by stimulation for 2 h with nigericin (5 μM). **C** Cell damage/death was assessed by quantifying the lactate dehydrogenase (LDH) content of the supernatant 12 h after LM infection. Data are presented as the mean ± SD of triplicates, and are representative of two independent experiments. *P < 0.05, **P < 0.01 by Student’s *t*-test. **D**, **E** WT or *Mint3*^–/–^ BMMFs were stained using a LIVE/DEAD Cell imaging kit 12 h after LM infection. Live (green) and dead (red) cells were discriminated using fluorescence microscopy. **D** Representative photographs. Scale bars = 100 μM. **E** Quantification of the ratio of dead cells in total cells. *n* = 3 per group. *P < 0.05, **P < 0.01 by Student’s *t*-test.

ROS and NO play important roles in the intracellular killing of LM in macrophages^5^. BMMFs were, therefore, treated with various infectious doses of LM to determine their effects on the production of intracellular ROS and NO. Unexpectedly, *Mint3*^–/–^ BMMFs produced less ROS (Fig. 3A) and comparable NO (Fig. 3B) relative to WT BMMFs. Thus, the decreased LM growth within *Mint3*^–/–^ macrophages was not attributed to bacterial killing by increased ROS and NO.

Next, we examined whether Mint3 depletion affects pyroptosis and thereby suppresses LM growth within macrophages. The amount of LDH released from damaged macrophages in the culture supernatant revealed that LM infection was more cytotoxic to *Mint3*^–/–^ BMMFs (Fig. 3C). Furthermore, imaging analyses showed that LM-infected *Mint3*^–/–^ BMMFs had a higher death rate than LM-infected WT BMMFs (Fig. 3D, E). Therefore, Mint3 depletion promoted pyroptosis in LM-infected macrophages.

### Mint3 deficiency promotes inflammasome activation in macrophages

**Fig. 4:**
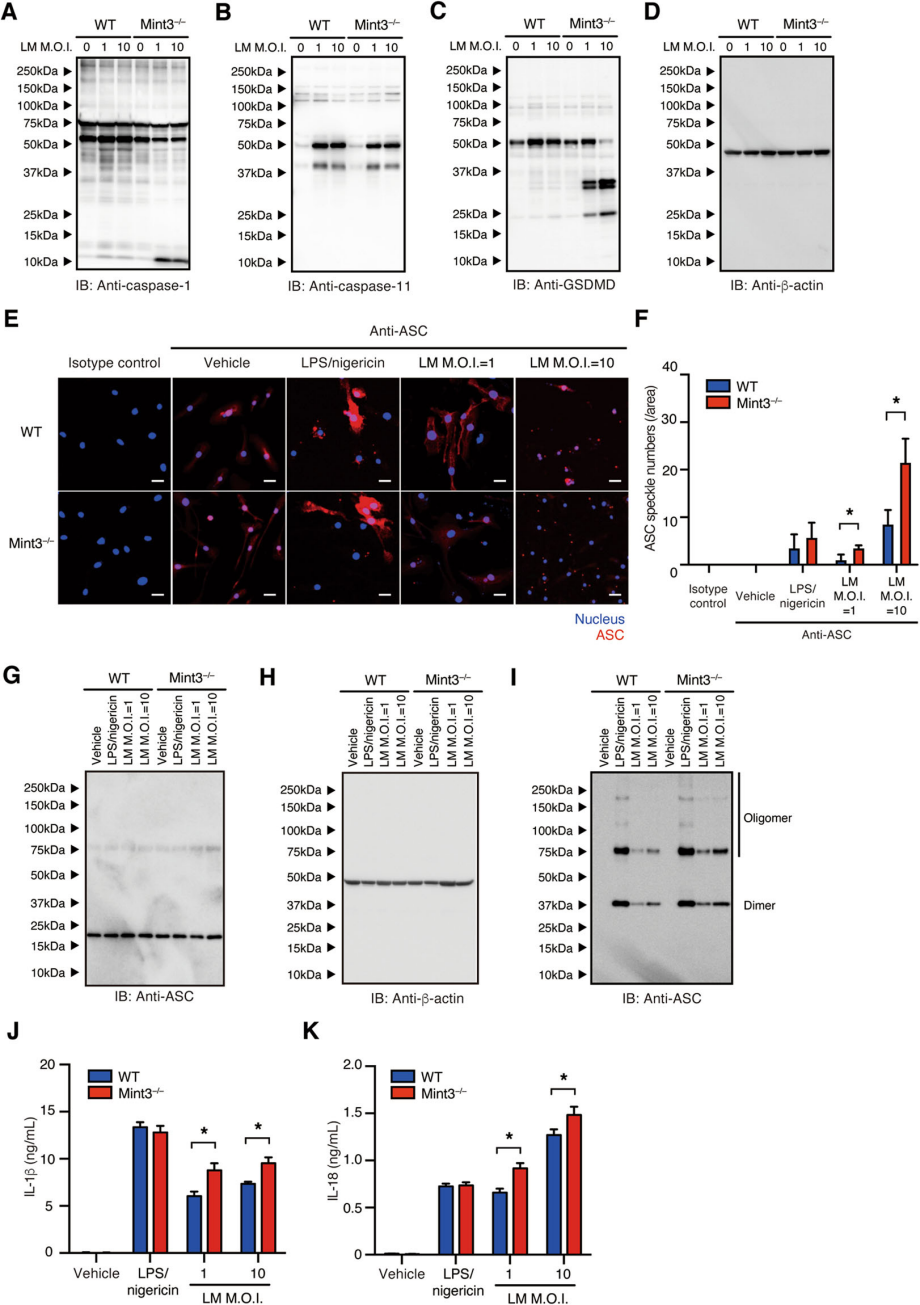
Mint3 depletion promotes inflammasome activation in LM-infected macrophages. **A–D** Inflammasome activation in WT or *Mint3*^–/–^ BMMFs infected in vitro with LM (MOI = 1 or 10). Caspase-1 (**A**), caspase-11 (**B**), or gasdermin D (GSDMD) (**C**) expression in the total cell lysate of WT or *Mint3*^–/–^ BMMFs after LM infection was measured. Expression of β-actin (**D**) was used as a loading control. **E** ASC speckle formation in WT or *Mint3*^–/–^ BMMFs stained with an anti-mouse ASC antibody 12 h after LM infection. The nuclei (blue) and ASC (red) were distinguished via fluorescence microscopy. Scale bars = 50 μM. **F** Quantification of ASC-specific speckles, *n* = 4 per group. *P < 0.05 by Student’s *t*-test. **G–I** Immunoblot analysis of **G** the total cell lysate or **I** disuccinimidyl-suberate-treated Triton-insoluble fractions in WT or *Mint3*^–/–^ BMMFs after LM infection. **H** Expression of β-actin in the total cell lysate was used as a loading control. IL-1β (**J**) and IL-18 (**K**) levels in the supernatants of the cultures of WT and *Mint3*^–/–^ BMMFs. Data are presented as the means ± SDs of triplicates. Data are representative of two independent experiments. *P < 0.05 by the Student’s *t*-test.

When LM infects macrophages, genomic DNA and LLO from LM act as stimulators to activate host AIM2 or NLRP3 inflammasomes. Furthermore, activated inflammasomes promote caspase-1-dependent cleavage of GSDMD^3,9–12,32^. The N-terminal fragment of GSDMD forms a polymeric pore in the cell membrane, causing pyroptosis of macrophages^8,13^. Thus, we next investigated whether the inflammasome was activated in *Mint3*^–/–^ macrophages. Immunoblotting analysis revealed that caspase-1, but not the structurally similar caspase-11, was activated more in LM-infected *Mint3*^–/–^ BMMFs than in LM-infected WT BMMFs (Fig. 4A, B). In parallel to the activation of caspase-1, the processing of GSDMD was promoted in *Mint3*^–/–^ BMMFs (Fig. 4C, D).

Upon activation of the inflammasome, one of its components, ASC, clusters to form a speckled structure^32^. Confocal fluorescence microscopy revealed an increased assembly of ASC in LM-stimulated *Mint3*^–/–^ BMMFs, as indicated by more numerous ASC-specific speckles than in WT BMMFs (Fig. 4E, F). Despite the similar expression levels of ASC in the total cell lysates (Fig. 4G, H), the immunoblotting analysis results revealed more ASC dimers or oligomers in the Triton X-100-insoluble fraction of the LM-stimulated *Mint3*^–/–^ BMMFs than that of the LM-stimulated WT BMMFs (Fig. 4I). Furthermore, the levels of IL-1β (Fig. 4J) and IL-18 (Fig. 4K) in the culture supernatants 24 h after LM infection were significantly increased in LM-infected *Mint3*^–/–^ BMMFs. These findings indicate that Mint3 deficiency activated inflammasomes and efficiently induced pyroptosis in LM-infected macrophages.

### Inhibition of glycolysis promotes pyroptosis and confers resistance to LM infection in macrophages

**Fig. 5:**
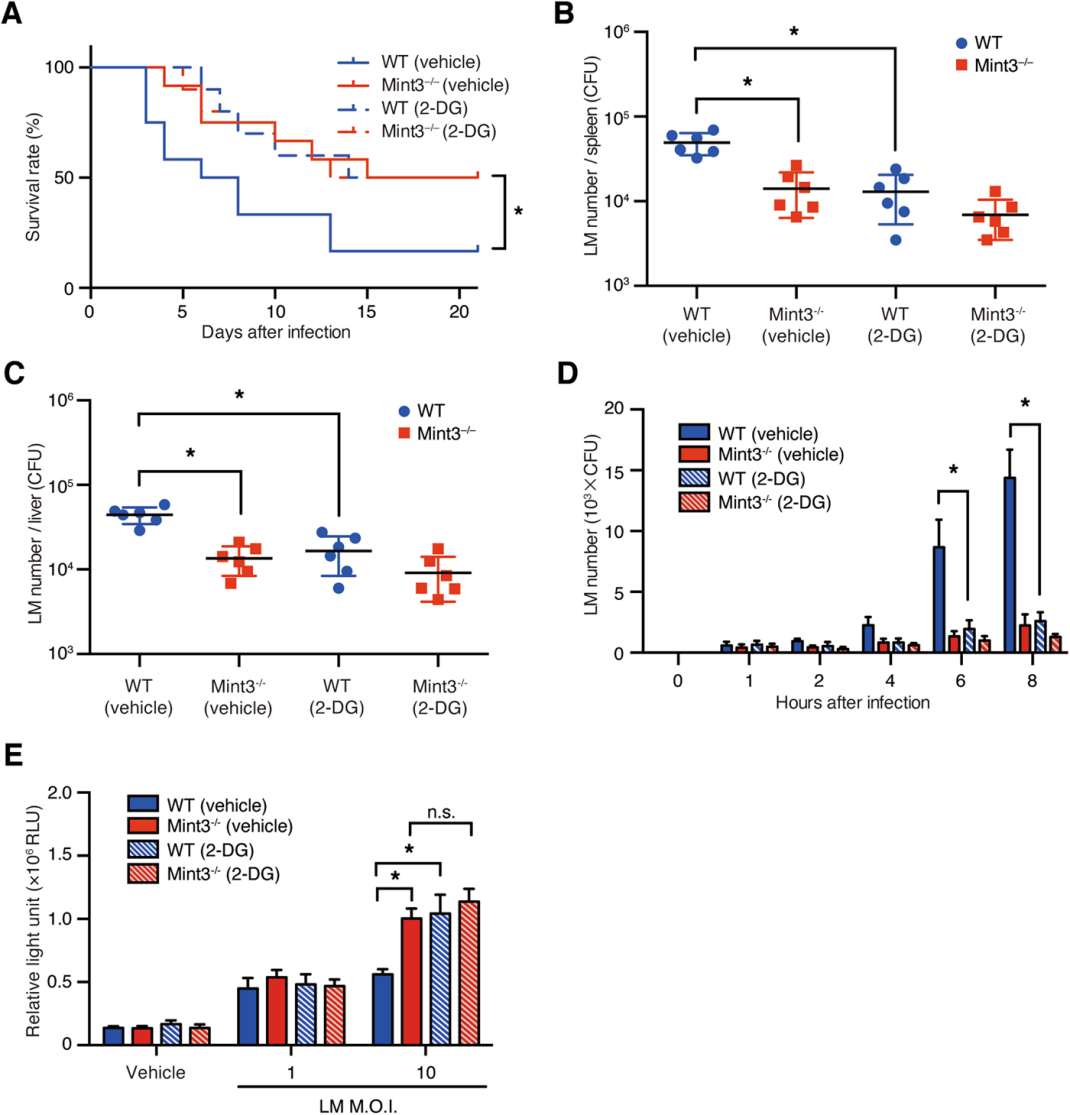
Glycolysis inhibition enhances resistance to LM infection. **A–C** Influence of 2-deoxy-d-glucose (2-DG) on LM infection in vivo. **A** WT and *Mint3*^–/–^ mice (*n* = 12 per group) were infected by intraperitoneal injection of 2 × 10^5^ CFUs of LM, followed by the assessment of survival rates. Each mouse received intraperitoneal pre-administration of control vehicle or 2-DG (500 mg/kg) 1 h prior to LM infection. Data are representative of two independent experiments. *P < 0.05 by the log-rank test. **B** The bacterial burdens in the spleen and **C** liver were counted on days 1, 3, and 7 after LM infection. Data are presented as the means ± SEMs and are representative of two independent experiments. *P < 0.05 by the Mann–Whitney *U* test. **d** BMMFs from WT or *Mint3*^–/–^ mice were infected in vitro with 10^5^ CFUs of LM (MOI = 1) after pretreatment with either control vehicle or 2-DG (100 μg/mL) 1 h before LM infection. The bacterial burdens were determined 1, 2, 4, 6, and 8 h after infection. Data are presented as the means ± SDs of triplicates. Data are representative of two independent experiments. *P < 0.05 by the Student’s *t*-test. **E** The cell deaths in LM-infected WT and *Mint3*^–/–^ BMMFs treated with vehicle or 2-DG were assessed by quantifying the lactate dehydrogenase (LDH) contents of the supernatants 12 h after LM infection. Data are presented as the mean ± SD of triplicates and are representative of two independent experiments. *P < 0.05 by Student’s *t*-test.

*Mint3*^–/–^ macrophages exhibit defects in ATP production via glycolysis^22,23^. We, therefore, investigated whether inhibition of glycolysis altered host resistance to LM infection. For this purpose, we administered the glycolysis inhibitor 2-deoxyglucose (2-DG) intraperitoneally into WT and *Mint3*^–/–^ mice before LM infection. The survival rate of 2-DG-administrated WT mice recovered to the same level as that in *Mint3*^–/–^ mice (Fig. 5A). Moreover, 2-DG administration also reduced the LM burdens in the spleen (Fig. 5B) and liver (Fig. 5C) of WT mice 1 day after LM infection to the same levels as in *Mint3*^–/–^ mice. Furthermore, WT and *Mint3*^–/–^ BMMFs were infected with LM in the presence of 2-DG, and the intracellular bacterial burdens were analyzed. 2-DG decreased the intracellular proliferation of LM in WT BMMFs to levels comparable to those in *Mint3*^–/–^ BMMFs (Fig. 5D). However, 2-DG itself did not affect the replication of LM in growth media (Supplementary Fig. 2), consistent with published data^33^. Next, we examined whether 2-DG promotes pyroptosis in LM-infected macrophages. 2-DG alone did not induce cell death in BMMFs; however, 2-DG increased the death of LM-infected WT BMMFs to the levels of *Mint3*^–/–^ BMMFs (Fig. 5E). Taken together, Mint3 depletion promotes pyroptosis of LM-infected macrophages via attenuated glycolysis and thereby suppresses intracellular proliferation of LM in macrophages.

### Mint3-mediated glycolysis and ROS production control inflammasome activation in LM-infected macrophages

**Fig. 6:**
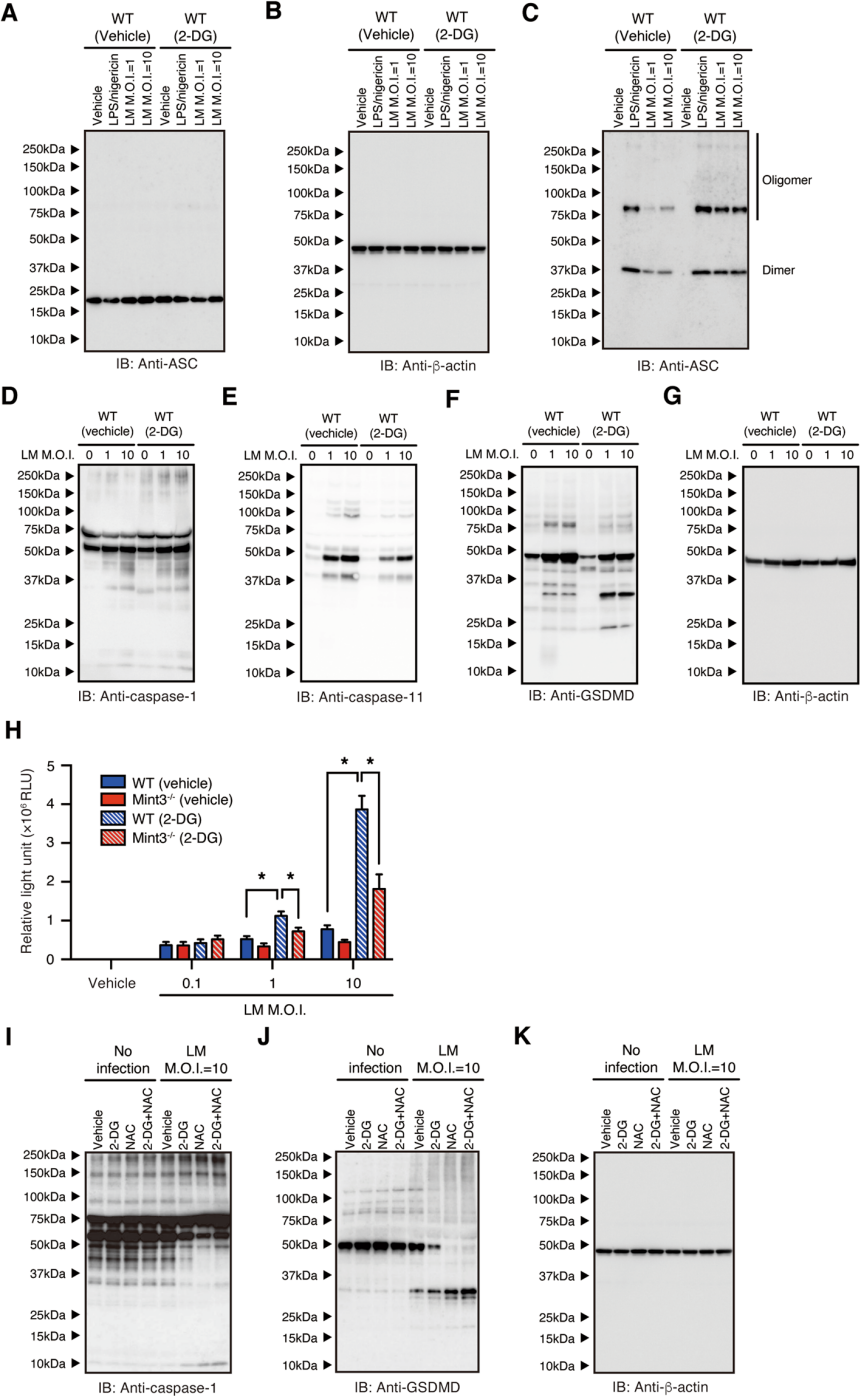
Mint3-mediated glycolysis and ROS production control inflammasome activation in LM-infected macrophages. **A–C** Immunoblot analysis of the **A** total cell lysate or **C** disuccinimidyl-suberate-treated Triton-insoluble fractions in LM-infected WT BMMFs treated with vehicle or 2-DG. **B** The expression of β-actin in the total cell lysate was used as a loading control. **D–G** Inflammasome activation in 2-DG-treated WT or *Mint3*^–/–^ BMMFs, which were infected in vitro with LM (MOI = 1 or 10). **D** Caspase-1, **E** caspase-11, or **F** GSDMD expression was measured in the total cell lysates of LM-infected WT BMMFs treated with vehicle or 2-DG. **G** Expression of β-actin was used as a loading control. **H** ROS production in 2-DG-treated WT or *Mint3*^–/–^ BMMFs. Production of ROS was measured via luminometry 8 h after LM infection. Data are presented as the means ± SDs of triplicates. Data are representative of two independent experiments. *P < 0.05 by the Student’s *t*-test. **I–K** Inflammasome activation in LM-infected WT BMMFs treated with vehicle, 2-DG, *N*-acetyl-l-cystine (NAC), or 2-DG + NAC. **I** Caspase-1 and **J** GSDMD expression was measured in the total cell lysates of LM-infected WT BMMFs treated with vehicle or 2-DG. **K** Expression of β-actin was used as a loading control.

Glycolysis inhibition promoted pyroptosis in LM-infected macrophages. Thus, whether glycolysis controls inflammasome activation was further examined. 2-DG treatment promoted ASC polymerization in LM-infected BMMFs without affecting ASC levels (Fig. 6A–C). In parallel with increased ASC polymerization, 2-DG treatment also promoted caspase-1 activation and GSDMD processing in LM-infected BMMFs (Fig. 6D–G). Thus, glycolysis suppressed inflammasome activation in LM-infected macrophages. Even though 2-DG treatment promoted caspase-1 activation and GSDMD processing, these changes were moderate compared with Mint3 depletion (Fig. 4A, C), suggesting another mechanism by which Mint3 controls inflammasome activation and pyroptosis.

Mint3 depletion decreased ROS production in LM-infected macrophages (Fig. 3A). Therefore, we examined whether glycolysis inhibition also suppressed ROS production in LM-infected WT and *Mint3*^–/–^ BMMFs. Interestingly, 2-DG treatment increased ROS production in LM-infected BMMFs, and *Mint3*^–/–^ BMMFs still showed decreased ROS production (Fig. 6H). These results indicate that Mint3 deficiency attenuated ROS production in LM-infected macrophages independent of defects in glycolysis. ROS inactivates caspase-1 via oxidation^34^. Thus, we examined whether the combined inhibition of glycolysis and ROS induces prominent caspase-1 activation and GSDMD processing, similar to Mint3 deficiency in LM-infected macrophages. 2-DG or the ROS scavenger NAC treatment partially activated caspase-1 and induced GSDMD processing in LM-infected BMMFs; meanwhile, the combination of 2-DG and NAC strikingly induced caspase-1 activation and GSDMD processing (Fig. 6I–K). Thus, Mint3-mediated glycolysis and ROS production coordinately control inflammasome activation and pyroptosis in LM-infected macrophages.

### Resistance to LM infection caused by Mint3 deficiency requires activated caspase-1

**Fig. 7:**
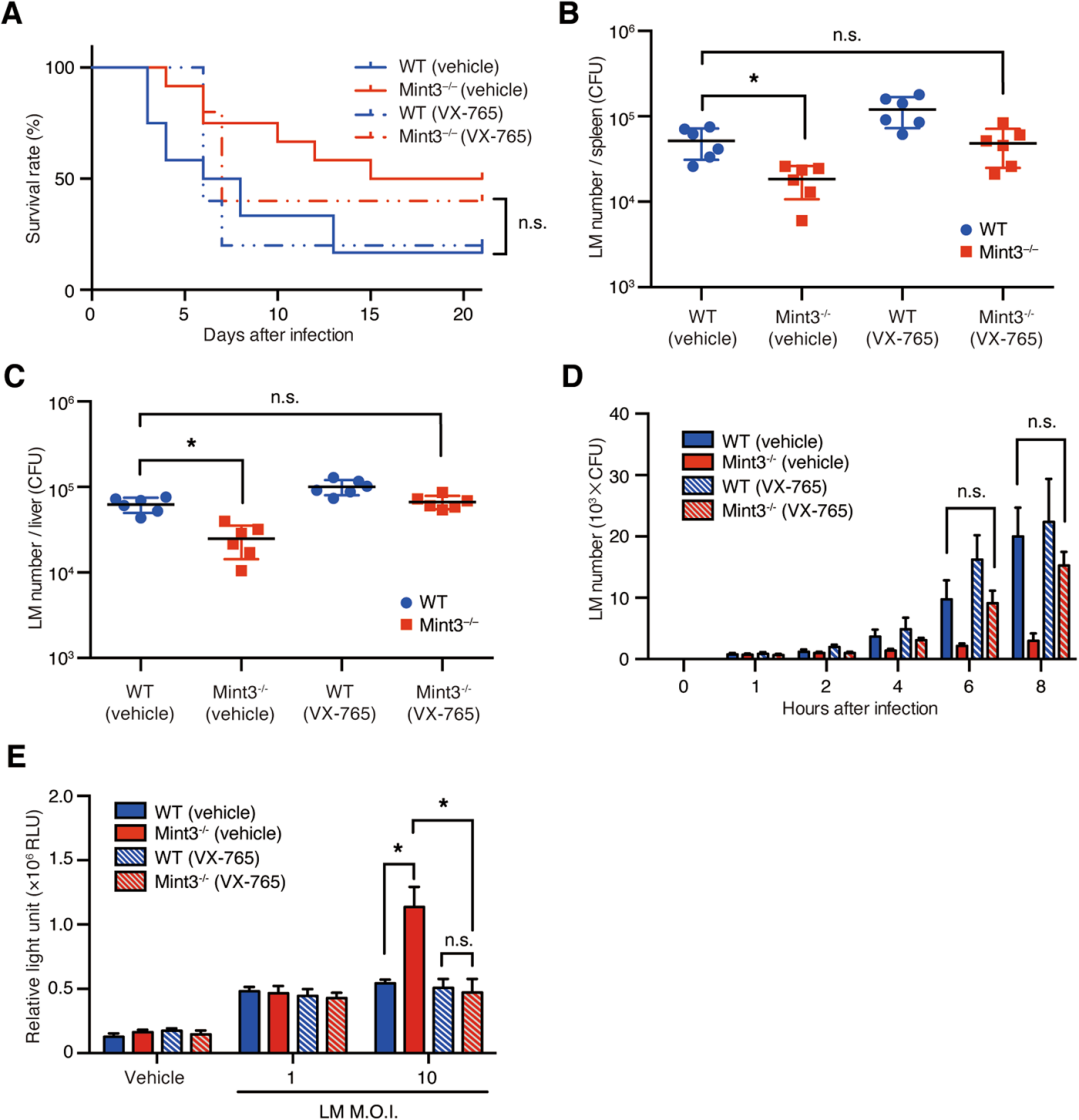
Resistance to LM infection caused by deficiency of Mint3 is mediated by a caspase-1-dependent mechanism. **A–C** Influence of the caspase-1 inhibitor VX-765 on LM infection in vivo. **A** WT and *Mint3*^–/–^ mice (*n* = 10 per group) were infected via intraperitoneal injection of 2 × 10^5^ CFUs of LM, followed by the assessment of survival rates. Each mouse received intraperitoneal pre-administration of either control vehicle or VX-765 (50 mg/kg) 1 h before LM infection. Data are representative of two independent experiments. *P < 0.05 by the log-rank test. **B** The bacterial burdens in the spleen and **C** liver were counted 1, 3, and 7 days after LM infection. Data are presented as the means ± SEMs and are representative of two independent experiments. *P < 0.05 by the Mann–Whitney *U* test. **D** Influence of VX-765 on LM infection in vitro. BMMFs from WT or *Mint3*^–/–^ mice were infected in vitro with 10^5^ CFUs of LM (MOI = 1), following pretreatment with either control vehicle or VX-765 (10 μM) 1 h before LM infection. The bacterial burdens were determined 1, 2, 4, 6, and 8 h after infection. Data are presented as the means ± SDs of triplicates. Data are representative of two independent experiments. *P < 0.05 by the Student’s *t*-test. **E** The cell deaths in LM-infected WT and *Mint3*^–/–^ BMMFs treated with VX-765 (10 μM) were assessed by quantifying the LDH contents of the supernatants 12 h after LM infection. Data are presented as the mean ± SD of triplicates and are representative of two independent experiments. *P < 0.05 by Student’s *t*-test.

Since Mint3 depletion rapidly activated the inflammasome and induced pyroptosis of LM-infected macrophages, we investigated whether inhibition of caspase-1 early during LM infection altered resistance to LM infection. For this purpose, WT and *Mint3*^–/–^ mice were administered with the potent and selective caspase-1 inhibitor (VX-765)^35^ and then infected with LM. Under these conditions, the survival rate of *Mint3*^–/–^ mice was almost equivalent to that of WT mice (Fig. 7A). Moreover, 1 day after the infection, the bacterial burdens in the spleen (Fig. 7B) and liver (Fig. 7C) of *Mint3*^–/–^ mice were increased to the same levels as in WT mice. When WT and *Mint3*^–/–^ BMMFs were infected in the presence of VX-765, the suppression of intracellular LM growth and enhancement of cell death in *Mint3*^–/–^ BMMFs were abolished (Fig. 7D, E). Taken together, these results indicate that the resistance of *Mint3*^–/–^ mice to LM infection requires activated caspase-1.

## Discussion

**Fig. 8:**
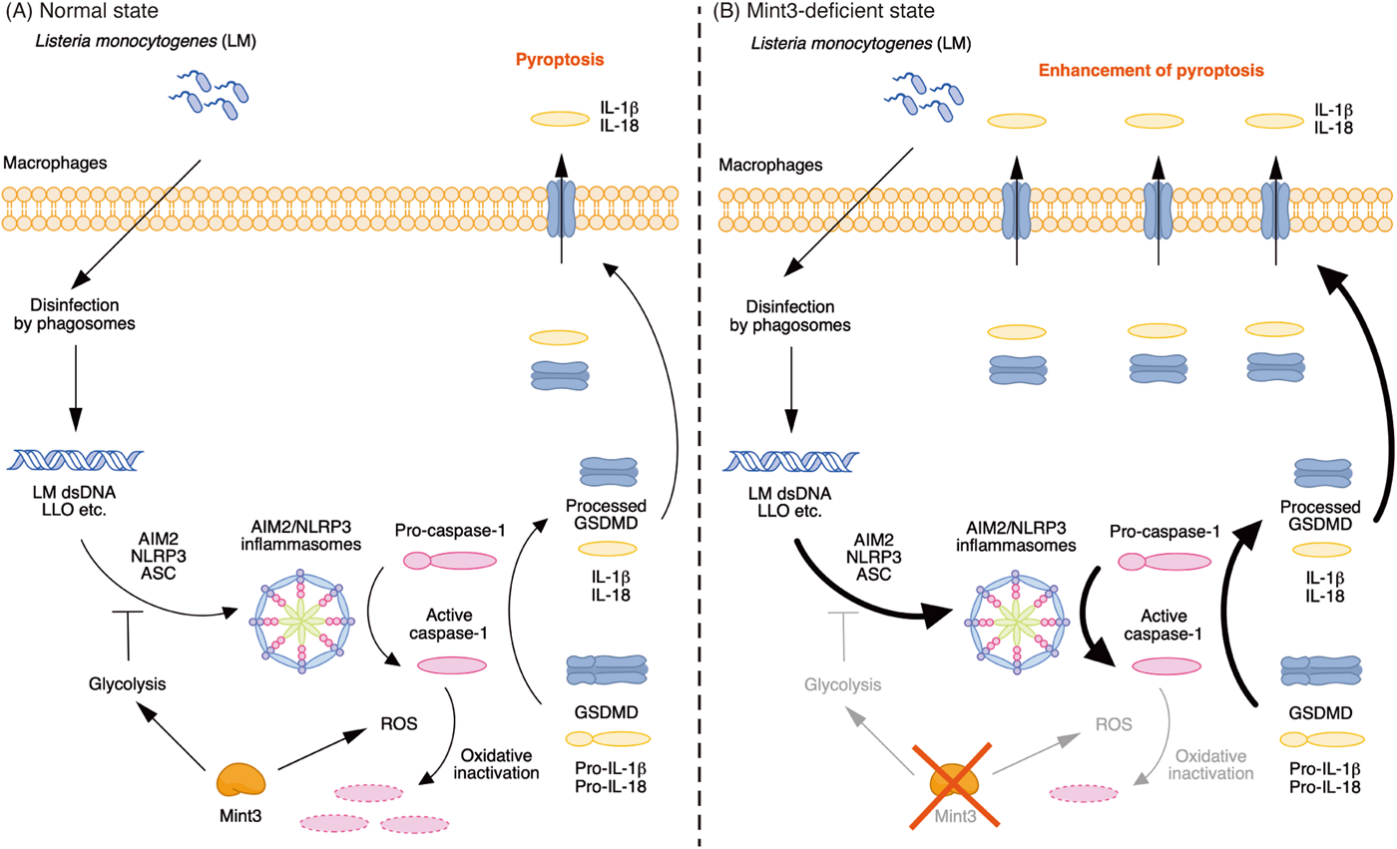
Mint3 controls inflammasome activation and pyroptosis in LM-infected macrophages. (**A**) Mint3-mediated glycolysis inhibits ASC aggregation and the resulting inflammasome activation during LM infection. In addition, Mint3-mediated production of reactive oxygen species also inactivates caspase-1. Thus, Mint3 suppresses caspase-1-dependent GSDMD processing and pyroptosis in macrophages during LM infection. (**B**) When Mint3 is depleted, inflammasome activation and pyroptosis are enhanced in macrophages during LM infection, thereby serving to attenuate LM proliferation in macrophages and the associated listeriosis.

Here, we show that a Mint3-mediated pathway contributes to severe listeriosis in mice. Specifically, caspase-1-dependent processing of GSDMD was enhanced in LM-infected macrophages isolated from *Mint3*^–/–^ mice, accompanied by strong induction of pyroptosis. In LM-infected macrophages, Mint3 depletion promoted pyroptosis and LM clearance by suppressing glycolysis-dependent inflammasome inactivation and ROS-dependent caspase-1 inactivation. Therefore, bacterial clearance was enhanced after LM infection, and the survival rate of *Mint3*^–/–^ mice after the infection was increased compared with that in WT mice (Fig. 8). Together, these findings suggest that Mint3 may function as a negative regulator of inflammasome activation and pyroptosis during LM infection by integrating glycolysis and ROS production.

*Mint3*^–/–^ mice were resistant to LM infection (Fig. 1A). The bacterial burdens in organs after infection with LM in *Mint3*^–/–^ mice became lower on day 1 of infection, in contrast to WT mice (Fig. 1B). The levels of IL-1β and IL-18 induced by inflammasome activation were significantly higher in *Mint3*^–/–^ mice on day 3 of infection (Fig. 1C, D). These findings suggest that LM infection activated the inflammasome earlier in *Mint3*^–/–^ mice, and LM-infected cells, which contributed to the spread of infection, efficiently underwent pyroptosis, leading to improved survival of *Mint3*^–/–^ mice. This idea is also supported by the finding that a single injection of the caspase-1 inhibitor VX-765 1 h before LM injection increased the LM burden in the liver and spleen and worsened the survival rate in *Mint3*^–/–^ mice to the levels of WT mice (Fig. 7A–C). In addition to pyroptosis, the defects of motility in *Mint3*^–/–^ macrophages^22,29,30^ may partially affect the facile translocation of the LM within macrophages from the abdominal cavity to other organs.

Regulation of inflammasome activation by glycolysis remains ambiguous and controversial^36^. For example, the glycolytic enzymes hexokinase (HK) 1 and pyruvate kinase isoform 2 (PKM2) promote inflammasome activation, whereas inhibitors of the glycolytic enzymes α-enolase and glyceraldehyde phosphate dehydrogenase promote inflammasome activation^37–39^. In our study, 2-DG, which inhibits hexokinase and phosphoglucoisomerase, promoted the activation of inflammasomes in LM-infected macrophages (Fig. 6). Because the proposed mechanisms of inflammasome activation by HK1 and PKM2 do not necessarily rely on the metabolic flux or the metabolites of glycolysis, these metabolic factors might suppress inflammasome activation, reflecting the data regarding glycolytic inhibitors. Mint3 promotes glycolysis-related genes by activating HIF-1 resulting in enhanced glycolysis in macrophages^22,29^. Enhanced glycolysis by Mint3 suppressed inflammasome activation and pyroptosis in LM-infected macrophages. Since 2-DG treatment did not further promote pyroptosis in LM-infected *Mint3*^–/–^ macrophages (Fig. 5E), Mint3-mediated inflammasome suppression might also depend on the metabolic flux and/or metabolites of glycolysis rather than the expression of glycolytic enzymes.

In addition to inflammasome activation, Mint3 promoted ROS levels resulting in oxidative inactivation of caspase-1 and decreased pyroptosis in LM-infected macrophages. ROS-induced oxidation of caspase-1 reduces the activity of this protein in *Sod1*^–/–^ mice^34^. In corroboration, NAC treatment increased active caspase-1 in LM-infected WT macrophages (Fig. 6I). Mint3-mediated ROS production in LM-infected macrophages was independent of glycolysis (Fig. 6H). Mint3 can activate the HIF-1 and NF-κB signaling pathways in macrophages^26^, and both HIF-1 and NF-κB can induce pro-oxidant genes, including *NOX2* (refs. ^40,41^). Thus, Mint3 might promote the expression of pro-oxidant genes and result in ROS production via the HIF-1 and/or NF-κB signaling pathways independent of glycolysis in LM-infected macrophages.

In contrast to the LM infection, LPS and nigericin induced inflammasome activation and IL-1β/IL-18 secretion in *Mint3*^–/–^ macrophages at the same levels as in WT cells (Fig. 4E–K). Thus, the regulation of inflammasome activation and pyroptosis by Mint3 probably depends on the stimulus. LM mainly activates the AIM2 inflammasome^9,10^. On the other hand, flagellin of bacteria or the type III secretion system of Gram-positive bacteria activate the NLRC4 inflammasome, and cytosolic LPS of Gram-negative bacteria directly activates caspase-11, which promotes GSDMD cleavage and pyroptosis^42^. Whether Mint3 also controls other pathways of inflammasome activation and pyroptosis needs to be addressed in future studies. In the context of LM infection, Mint3 exacerbated the disease by suppressing inflammasomes and pyroptosis in macrophages. Interestingly, inflammasome and pyroptosis have been implicated in the progression of degenerative disorders, such as amyotrophic lateral sclerosis, Alzheimer’s disease, and Parkinson’s disease, and inflammatory diseases, such as periodic fever syndrome^43^. Thus, Mint3-mediated suppression of inflammasomes and pyroptosis in macrophages may play the beneficial roles in these diseases.

In conclusion, Mint3 expressed by macrophages contributes to severe listeriosis. Thus, inhibition of Mint3 may serve as a strategy to prevent the spread of LM infection and severe listeriosis by promoting pyroptosis.

## Materials and methods

### Mice

*Mint3*^–/–^ mice have previously been described^22^ (Riken Center for Developmental Biology Accession No. CDB0589K; http://www2.clst.riken.jp/arg/mutant%20mice%20list.html). These mice were backcrossed ≥10 times with C57BL/6 mice (CLEA Japan, Inc., Tokyo, Japan). The mice were housed under specific pathogen-free conditions. The animal experiments was conducted according to the protocol approved by the president of Kitasato University after the review by the Institutional Animal Care and Use Committee.

### Bacteria

Dr. Kikuo Nomoto (Medical Institute of Bioregulation, Kyushu University, Fukuoka, Japan) provided an LM EGD strain, which was cultured at 37 °C on tryptic soy agar (TSA) plates containing 0.4% d-glucose.

### Reagents

LPS from *Escherichia coli* 0111:B4, nigericin, and 2-DG [Sigma-Aldrich (St. Louis, MO)]; *N*-acetyl-L-cysteine (NAC) [Nacalai Tesque (Kyoto, Japan)]; and Belnacasan (VX-765) [Invivogen (San Diego, CA)] were purchased.

### LM infection of mice

Sex- and age-matched C57BL/6 and *Mint3*^–/–^ mice were intraperitoneally injected with 2 × 10^5^ CFUs (unless otherwise indicated) of LM, followed by the assessment of survival rates. Clinical scoring was performed only on surviving mice, based on a previously reported scoring list for a pneumococcal meningitis mouse model^44,45^. Three items in the list were evaluated: weight loss, activity, and state of fur coat. The range of each scoring parameter was from 0 (no change) to 10 (maximum score), and the changes in each mouse from day 0 to day 7 were plotted in a graph. Organs were lysed and homogenized with sterile water containing 0.2% Triton X-100. Organ lysates were diluted as required and plated onto TSA plates. After incubation at 37 °C overnight, the CFUs per organ were counted. For chemical treatments, C57BL/6 and *Mint3*^–/–^ mice were intraperitoneally administered 2-DG (500 mg/kg) or VX-765 (50 mg/kg) for 1 h before infection with LM.

### Preparation of macrophages and LM stimulation in vitro

TG-MFs were prepared as previously described^46^. BMMFs were prepared by culturing bone marrow cells for 5–8 days in RPMI1640 medium supplemented with 10% fetal bovine serum and antibiotics (100 IU/mL penicillin and 100 μg/mL streptomycin) containing M-CSF (25 ng/mL, Peprotech, Rocky Hill, NJ). For MF stimulation, MFs (1 × 10^5^) were seeded in 24-well culture plates (Corning, Corning, NY) and incubated overnight. TG-MFs and BMMFs were infected with the indicated doses of LM, and 100 μg/mL gentamicin sulfate (Sigma–Aldrich) was added 1 h after infection to kill the extracellular bacteria. MFs were lysed and homogenized in the lysis buffer, and the lysates were diluted as required and plated onto TSA plates. CFUs were counted after incubation at 37 °C overnight. BMMFs were primed for 6 h with LPS (50 ng/mL) and stimulated with nigericin (5 μM) for 2 h. For chemical reagent treatments, BMMFs were incubated with 2-DG (100 μg/mL), NAC (1 mM), or VX-765 (10 μM) for 1 h before infection with LM.

### Cross-linking

ASC components were cross-linked as previously described^32^ and then collected with centrifugation for 15 min at 6000 × *g*. The pellets were dissolved in Laemmli sample buffer.

### Low-carbon-source medium

LM was cultured in a low-carbon-source medium as previously described^33^.

### Statistical analysis

Statistical analyses were performed using the log-rank test and GraphPad Prism (GraphPad, San Diego, CA, USA). Survival curves were generated using the Kaplan–Meier method. The Mann–Whitney *U* test and Student’s *t*-test were used for evaluating the significance of differences between datasets, presented as mean ± SEM or SD; *P* values <0.05 were considered significant.

## Supplementary information

Supplementary Materials and Methods

Supplementary Figure 1

Supplementary Figure 2
