# Supplementary material for: Mint3 depletion-mediated glycolytic and oxidative alterations promote pyroptosis and prevent the spread of Listeria monocytogenes infection in macrophages
Source: Cell Death Dis. 2021 Apr 14;12(4):404. doi: 10.1038/s41419-021-03691-y (PMC8046764; doi:10.1038/s41419-021-03691-y)
Supplement: Supplementary file 1 — Supplementary Materials and Methods [file 41419_2021_3691_MOESM1_ESM.docx]

**Mint3 depletion-mediated glycolytic and oxidative alterations promote pyroptosis and prevent the spread of *Listeria monocytogenes* infection in macrophages**

**Takayuki Uematsu^1^*, Kohsuke Tsuchiya^2^, Noritada Kobayashi^1^, Motoharu Seiki^3^,**

**Jun-ichiro Inoue^4^, Shuichi Kaneko^5^**, **Takeharu Sakamoto^4,5^***

^1^Biomedical Laboratory, Division of Biomedical Research, Kitasato University Medical Center, Arai, Kitamoto, Saitama, Japan

^2^Division of Immunology and Molecular Biology, Cancer Research Institute, Kanazawa University, Kakuma-machi, Kanazawa, Ishikawa, Japan

^3^Division of Cancer Cell Research, Institute of Medical Science, The University of Tokyo, Shirokanedai, Minato-ku, Tokyo, Japan

^4^Division of Cellular and Molecular Biology, Institute of Medical Science, The University of Tokyo, Shirokanedai, Minato-ku, Tokyo, Japan

^5^Department of System Biology, Institute of Medical, Pharmaceutical and Health Sciences, Kanazawa University, Takara-machi, Kanazawa, Ishikawa, Japan

*Correspondence and requests for materials should be addressed to T.U. or T.S. (email: T.U.;[tuematsu@insti.kitasato-u.ac.jp](mailto:tuematsu@insti.kitasato-u.ac.jp), T.S.; [t-saka@staff.kanazawa-u.ac.jp](mailto:t-saka@staff.kanazawa-u.ac.jp) ).

**Supplementary Information**

**Supplementary Materials and Methods**

**Mice**

*Mint3*^–/–^ mice have previously been described^1^ (Riken Center for Developmental Biology Accession No. CDB0589K; http://www2.clst.riken.jp/arg/mutant%20mice%20list.html). These mice were backcrossed ≥ 10 times with C57BL/6 mice (CLEA Japan, Inc., Tokyo, Japan). The mice were housed under specific pathogen-free conditions. The animal experiments was conducted according to the protocol approved by the president of Kitasato University after the review by the Institutional Animal Care and Use Committee.

**Bacteria**

Dr. Kikuo Nomoto (Medical Institute of Bioregulation, Kyushu University, Fukuoka, Japan) provided an LM EGD strain, which was cultured at 37 °C on tryptic soy agar (TSA) plates containing 0.4% D-glucose.

**Reagents**

LPS from *Escherichia coli* 0111:B4, nigericin, and 2-deoxyglucose (2-DG) [Sigma–Aldrich (St. Louis, MO)]; N-acetyl-L-cysteine (NAC) [Nacalai Tesque (Kyoto, Japan)]; and Belnacasan (VX-765) [Invivogen (San Diego, CA)] were purchased.

**LM infection of mice**

Sex- and age-matched C57BL/6 and *Mint3*^–/–^ mice were intraperitoneally injected with 2 × 10^5^ CFUs (unless otherwise indicated) of LM, followed by the assessment of survival rates. Clinical scoring was performed only on surviving mice, based on a previously reported scoring list for a pneumococcal meningitis mouse model^2,3^. Three items in the list were evaluated: weight loss, activity, and state of fur coat. The range of each scoring parameter was from 0 (no change) to 10 (maximum score), and the changes in each mouse from day 0 to day 7 were plotted in a graph. Organs were lysed and homogenized with sterile water containing 0.2% Triton X-100. Organ lysates were diluted as required and plated onto TSA plates. After incubation at 37 °C overnight, the CFUs per organ were counted. For chemical treatments, C57BL/6 and *Mint3*^–/–^ mice were intraperitoneally administered 2-DG (500 mg/kg) or VX-765 (50 mg/kg) for 1 h before infection with LM.

**Preparation of macrophages (MFs) and LM stimulation *in vitro***

Thioglycolate-induced peritoneal macrophages (TG-MFs) were prepared as previously described^1^. Bone marrow-derived macrophages (BMMFs) were prepared by culturing bone marrow cells for 5–8 days in RPMI1640 medium supplemented with 10% fetal bovine serum and antibiotics (100 IU/mL penicillin and 100 μg/mL streptomycin) containing M-CSF (25 ng/mL, Peprotech, Rocky Hill, NJ). For MF stimulation, MFs (1 × 10^5^) were seeded in 24-well culture plates (Corning, Corning, NY) and incubated overnight. TG-MFs and BMMFs were infected with the indicated doses of LM, and 100 μg/mL gentamicin sulfate (Sigma–Aldrich) was added 1 h after infection to kill the extracellular bacteria. MFs were lysed and homogenized in the lysis buffer, and the lysates were diluted as required and plated onto TSA plates. CFUs were counted after incubation at 37 °C overnight. BMMFs were primed for 6 h with LPS (50 ng/mL) and stimulated with nigericin (5 μM) for 2 h. For chemical reagent treatments, BMMFs were incubated with 2-DG (100 μg/mL), NAC (1 mM), or VX-765 (10 μM) for 1 h before infection with LM.

**Cytokine assays**

Peritoneal washes of LM-infected mice and cell culture supernatants from TG-MFs or BMMFs were assayed using ELISA kits specific for IL-1β (BioLegend, San Diego, CA) or IL-18 (MBL, Nagoya, Japan). All measurements were performed in triplicate.

**ROS and NO assays**

BMMFs were infected with LM at a multiplicity of infection (M.O.I.) = 0.1, 1, or 10 for the indicated period. The levels of ROS in BMMFs and NO in culture supernatants were quantitated using the ROS-Glo H_2_O_2_ Assay and Griess Reagent System, respectively, according to the manufacturer’s instructions (Promega, Madison, WI).

**Assessment of cell death**

BMMFs were infected with LM at an MOI = 1 or 10 for 12 h. Culture supernatants were collected after infection and assayed for lactate dehydrogenase (LDH) activity using an LDH-Glo Cytotoxicity Assay (Promega) according to the manufacturer’s instructions. The numbers of living and dead BMMFs were measured using a LIVE/DEAD Cell Imaging Kit (Life Technologies) according to the manufacturer’s instructions, and images were captured using a ZOE Fluorescent Cell Imager (Bio-Rad Laboratories, Hercules, CA).

**Immunoblotting**

Total cell lysates and Triton-insoluble fractions were separated using SDS-PAGE, electrophoretically transferred onto PVDF membranes and subjected to immunoblotting using rabbit antibodies against pro-caspase1+p10+p12, caspase11, GSDMD (clones EPR16883, EPR18628, and EPR19828, respectively; Abcam, Cambridge, United Kingdom); and ASC (clone D2W8U; Cell Signaling Technology, Danvers, MA); or a mouse anti-β-actin antibody (clone C4; Merck Millipore, Burlington, MA).

**Immunofluorescence**

BMMFs (2 × 10^5^ cells per well) seeded in eight-well chamber plates were washed twice, fixed in 4% paraformaldehyde, and permeabilized with 0.1% Triton X-100. BMMFs were incubated with a rabbit anti-ASC antibody (clone D2W8U, Cell Signaling) and then with an Alexa 555-labeled IgG (H+L) F(ab')2 fragment specific for rabbit IgG (4413, Cell Signaling). Nuclei were stained with Hoechst 33342 (Life Technologies). BMMFs were visualized using confocal microscopy (Carl Zeiss, Oberkochen, Deutschland)**.** Quantification of ASC speckles was performed by using ImageJ software (National Institute of Health).

**Cross-linking**

ASC components were cross-linked as previously described^2^ and then collected with centrifugation for 15 min at 6,000 × *g*. The pellets were dissolved in Laemmli sample buffer.

**Low-carbon-source medium**

LM was cultured in a low carbon-source medium as previously described^3^.

**Statistical analysis**

Statistical analyses were performed using the log-rank test and GraphPad Prism (GraphPad, San Diego, CA, USA). Survival curves were generated using the Kaplan–Meier method. The Mann–Whitney *U* test and Student *t*-test were used for evaluating the significance of differences between datasets, presented as mean ± SEM or SD; *P*-values < 0.05 were considered significant.

**References**

1. Hara T, Mimura K, Abe T, Shioi G, Seiki M, Sakamoto T. Deletion of the Mint3/Apba3 Gene in Mice Abrogates Macrophage Functions and Increases Resistance to Lipopolysaccharide-induced Septic Shock. *J. Biol. Chem.* **286**, 32542–32551 (2011)

2. Mook-Kanamori B, Geldhoff M, Troost D, van der Poll T, van de Beek D. Characterization of a *pneumococcal meningitis* mouse model. *BMC Infect. Dis.* **12**, 71 (2012)

3. Koopmans MM, Engelen-Lee JY, Brouwer MC, Jaspers V, Man WK, Vall Seron M *et al.* Characterization of a *Listeria monocytogenes* meningitis mouse model. *J. Neuroinflammation* **15**, 1–11 (2018)
