# Supplementary material for: Mint3 depletion-mediated glycolytic and oxidative alterations promote pyroptosis and prevent the spread of Listeria monocytogenes infection in macrophages
Source: Cell Death Dis. 2021 Apr 14;12(4):404. doi: 10.1038/s41419-021-03691-y (PMC8046764; doi:10.1038/s41419-021-03691-y)
Supplement: Supplementary file 2 — Supplementary Figure 1 [file 41419_2021_3691_MOESM2_ESM.docx]

**Supplementary Figure 1. Clinical score of LM-infected mice.** WT (n = 8 per group) and Mint3^–/–^ mice (n = 7 per group) were infected via intraperitoneal injection of 2 × 10^5^ CFUs of LM, followed by clinical assessment. Clinical scoring was performed only on surviving mice (one WT mouse died on day 6 and two WT mice died on day 7), based on a previously reported scoring list for a pneumococcal meningitis mouse model. Three items on the list were evaluated: weight loss, activity, and fur coat. The range of each scoring parameter was from 0 (no change) to 10 (maximum score), and the changes in each mouse from day 0 to day 7 were plotted in a graph. Data are presented as the means ± SEMs and are representative of two independent experiments. **P* < 0.05 by the Mann–Whitney *U* test.
