# Supplementary material for: Mint3 depletion-mediated glycolytic and oxidative alterations promote pyroptosis and prevent the spread of Listeria monocytogenes infection in macrophages
Source: Cell Death Dis. 2021 Apr 14;12(4):404. doi: 10.1038/s41419-021-03691-y (PMC8046764; doi:10.1038/s41419-021-03691-y)
Supplement: Supplementary file 3 — Supplementary Figure 2 [file 41419_2021_3691_MOESM3_ESM.docx]

**
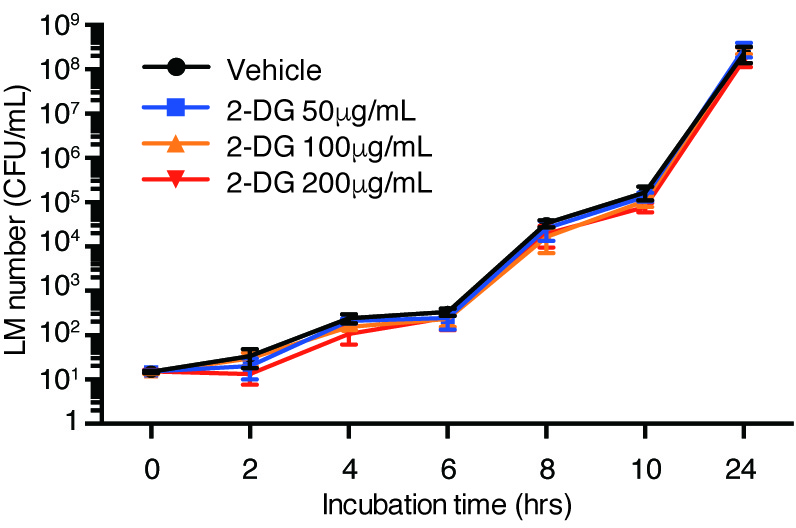
**

**Supplementary Figure 2. Growth curves of the LM EGD strain in low-carbon-source medium supplemented with 2-DG.** LM was cultured in a low carbon-source medium supplemented with 2-DG for 24 h. Data are presented as the means ± SD and are representative of two independent experiments. **P* < 0.05 by the Student’s *t* test.
